# Private-Sector Social Franchising to Accelerate Family Planning Access, Choice, and Quality: Results From Marie Stopes International

**DOI:** 10.9745/GHSP-D-15-00056

**Published:** 2015-06-12

**Authors:** Erik Munroe, Brendan Hayes, Julia Taft

**Affiliations:** ^a^​ Marie Stopes International, London, UK.

## Abstract

**Background:**

To achieve the global Family Planning 2020 (FP2020) goal of reaching 120 million more women with voluntary family planning services, rapid scale-up of services is needed. Clinical social franchising, a service delivery approach used by Marie Stopes International (MSI) in which small, independent health care businesses are organized into quality-assured networks, provides an opportunity to engage the private sector in improving access to family planning and other health services.

**Methods:**

We analyzed MSI’s social franchising program against the 4 intended outputs of access, efficiency, quality, and equity. The analysis used routine service data from social franchising programs in 17 African and Asian countries (2008–2014) to estimate number of clients reached, couple-years of protection (CYPs) provided, and efficiency of services; clinical quality audits of 636 social franchisees from a subset of the 17 countries (2011–2014); and exit interviews with 4,844 clients in 14 countries (2013) to examine client satisfaction, demographics (age and poverty), and prior contraceptive use. The MSI “Impact 2” model was used to estimate population-level outcomes by converting service data into estimated health outcomes.

**Results:**

Between 2008 and 2014, an estimated 3,753,065 women cumulatively received voluntary family planning services via 17 national social franchise programs, with a sizable 68% choosing long-acting reversible contraceptives (LARCs). While the number of social franchisee outlets increased over time, efficiency also significantly improved over time, with each outlet delivering, on average, 178 CYPs in 2008 compared with 941 CYPs in 2014 (*P* = .02). Clinical quality audit scores also significantly improved; 39.8% of social franchisee outlets scored over 80% in 2011 compared with 84.1% in 2014. In 2013, 40.7% of the clients reported they had not been using a modern method during the 3 months prior to their visit (95% CI = 37.4, 44.0), with 46.1% (95% CI = 40.9, 51.2) of them reporting having never previously used family planning at all. Analysis of age and poverty levels of clients indicate mixed results in bridging equity gaps: 57.4% of clients lived on under US$2.50/day in 2013 (95% CI = 54.9, 60.0) and 26.1% were 15–24 years old (95% CI = 23.8, 28.4), but only 15.1% lived on less than $1.25/day (95% CI = 13.8, 16.4) and 5.0% were 15–19 years old (95% CI = 3.9, 6.1). The services provided via social franchising are estimated to avert 4,958,000 unintended pregnancies and 7,150 maternal deaths.

**Conclusion:**

Social franchising through the existing private sector has the ability to rapidly scale-up access to high-quality family planning services, including LARCs, for the general population as well as young women and the poor, providing a promising model to help achieve the global FP2020 goal.

## Abstract (precis)

In just 7 years, Marie Stopes International (MSI) has scaled-up social franchising across Africa and Asia, from 7 countries to 17, cumulatively reaching an estimated 3.75 million clients including young adults and the poor. In 2014, 68% of clients chose long-acting reversible contraceptives, and many clients were adopters of family planning. Service quality and efficiency (couple-years of protection delivered per outlet) also improved significantly.

## Teaser

In just 7 years, Marie Stopes International (MSI) has scaled-up social franchising across Africa and Asia, from 7 countries to 17, cumulatively reaching an estimated 3.75 million clients including young adults and the poor. In 2014, 68% of clients chose long-acting reversible contraceptives, and many clients had not been using modern contraception in the past 3 months. Service quality and efficiency (couple-years of protection delivered per outlet) also improved significantly.

## INTRODUCTION

The London Summit on Family Planning in 2012 set a bold “FP2020” commitment to expand access to modern contraceptive methods for 120 million more women and girls by 2020.^1^ By 2013, an additional 8.4 million women and girls were using contraception in the 69 FP2020 focus countries. While the achievement represents an important milestone, progress must be greatly accelerated to reach the goal of 120 million by 2020.^1^

The private sector is a critical contributor to achieving the FP2020 goal, as a large proportion of the world’s population procures health care outside the public sector. Almost 60% of health care expenditures in the least-developed countries is spent in the private sector,^2^ and almost two-fifths of women using modern contraceptive methods report obtaining them from the private sector.^3^ While this represents a significant proportion of family planning service delivery, the quality of services provided by the private sector and the range of contraceptive method choices available are often limited.

Clinical social franchising is a service delivery approach in which small, independent health care businesses are organized into quality-assured networks. Social franchising involves intensive capacity building and support for providers, including clinical training, branding, quality monitoring, and commodity support, as well as marketing and demand generation among potential clients. In addition, franchising programs engage providers and clients through behavior change communication (BCC) and initiatives to ensure access for the lowest-income clients. Social franchising presents an opportunity to engage private providers in health care delivery to increase access to high-quality family planning and other services.^4^

There has been a large expansion in the number of clinical social franchising programs in the past 5 years, with an estimated 90 programs operating in low- and middle-income countries in 2013.^4^ Operational approaches and lessons learned from the clinical social franchising programs of Marie Stopes International (MSI) and Population Services International (PSI), two of the largest global franchisor entities, both of which experienced rapid growth in recent years, are described in a companion paper in *Global Health: Science and Practice*.^5^

An estimated 90 clinical social franchising programs are operating in low- and middle-income countries.

Despite the large increase in clinical social franchising programs, there is limited peer-reviewed published evidence of their health impact. A 2009 systematic review on social franchising found no studies eligible for inclusion in the review.^6^ More recently, a 2013 review that included gray as well as peer-reviewed literature identified 23 studies of 9 social franchising programs.^7^ The authors concluded that while social franchising has been shown to increase the number of clients and improve client satisfaction, there is limited and mixed evidence on whether clinical social franchising improves health care quality, equity, or population-level health outcomes.^7^ In addition, a recent technical consultation identified several evidence gaps related to the provision of voluntary long-acting reversible contraceptives (LARCs) and permanent methods of family planning via social franchising, including whether social franchising could reach new users of LARCs and the ability of social franchisees to provide permanent methods.^8^

The purpose of this paper is to present results from MSI’s social franchising programs through the end of 2014 to address key research gaps in global knowledge of social franchising, particularly the ability of social franchisees to increase access to LARCs and permanent methods as part of a broad method mix, to improve the quality of services provided, and to reach clients from groups that typically have high unmet need, such as those of lower-income levels.

MSI is one of the largest global providers of voluntary family planning services, delivering services in 2013 in 37 countries through 5 service delivery channels: static clinics, mobile outreach, social franchising, community-based distribution, and product social marketing.^9^ The international organization started its social franchising program in 2008 in 7 countries; by 2014, MSI was operating social franchising programs in 17 country programs across Africa and Asia.

MSI uses a fractional franchising model in which only selected franchisee services and commodities (typically, family planning for MSI franchising) are franchised. In other words, franchisees may provide other health services beyond family planning, but without MSI involvement and quality assurance. This fractional approach enables rapid scale-up of family planning services because it builds on existing service delivery mechanisms and infrastructure. More information on the operation of MSI’s fractional social franchising program, as well as the approaches used by PSI, can be found in the companion paper.^5^

## METHODS

### Data Sources

To assess the performance of the MSI social franchising program, we extracted data from 3 sources: routine program monitoring data, clinical quality audits, and client exit interviews. All 3 sources contained data from a small number of public-sector franchisees, which could not be removed readily and thus are included in all results shown. The proportion of facilities and services from the public sector were under 5% of those included in the analysis, and the results are considered to still be representative for private-sector franchising. All data collection and analysis were conducted according to international principles of maintaining privacy and confidentiality of personal information.

#### Routine Program Monitoring Data

Within country programs, social franchisees are required to maintain records of all family planning services provided. For short-acting contraceptive methods, such as pills and condoms, franchisees record the number of commodities distributed or sold to the end user, whereas for LARCs and permanent methods, they record each service provided (e.g., insertion, follow-up, removal). (Family planning counseling resulting in no service provision is also captured through routine data collection but was not used in this analysis.) Reporting procedures and formats (e.g., paper or electronic, weekly or monthly aggregated reporting, etc.) vary between countries, but at a minimum all franchisees periodically report the number of contraceptive commodities and services provided to the central support office in each country. This information is collected and aggregated at a national level, before being submitted electronically via Infor SunSystems (typically version 5.1 in early 2014 and version 6.1 by the end of the year) to the international MSI support office in London each month.

For this analysis, routine program monitoring data from January 1, 2008, to December 31, 2014, for the 17 MSI country programs operating social franchising were extracted and analyzed using Stata version 11. The 17 countries comprised 12 countries in Africa (Ethiopia, Ghana, Kenya, Madagascar, Malawi, Mali, Nigeria, Senegal, Sierra Leone, Uganda, Zambia, and Zimbabwe) and 5 countries in Asia (India, Pakistan, the Philippines, Vietnam, and Yemen).

#### Clinical Quality Audits

MSI globally oversees and operates a quality technical assistance (QTA) program with an annual external assessment to ensure country programs maintain a high standard of clinical quality and service delivery throughout their service delivery channels. The external audit complements the continuous review process, including internal audits conducted by the country program. External audits are carried out by global MSI medical advisors or approved consultants of clinical social franchisee outlets that have been operating as franchisee clinics for at least 12 months.

Using a standard checklist, auditors assess clinical governance, client focus, infection prevention, medical emergency management, management of equipment and supplies, and provision of core sexual and reproductive health services including family planning. Each checklist item is scored between 0 and 2, and each section is scored as the sum of all component scores out of the maximum possible score. An overall compliance score is calculated as the mean of the scores from each quality component assessed. The results of the QTA are used as part of ongoing program monitoring at a country and global level, as well as to inform improvements to services where required.

External audits of social franchisees commenced in 2011 for 9 country programs, but sampling size varied by country. Consequently, MSI standardized sampling approaches such that a sample of 10% of franchisees, with a maximum of 25, was audited. In 2011, only 3 country programs achieved this level of randomly selected social franchisee outlets. In 2012, after standardizing the sampling guidance, 10 country programs met the sampling requirements; 15 countries qualified in 2013; and 14 countries achieved the minimum sample of facilities in 2014.

QTA results for social franchisees are entered into a Microsoft Excel-based tool, which guides the QTA visit and is completed by the assessor while in-country. The final completed Excel tool, which automatically generates scores, is subsequently sent to the support office in London, where data across programs are managed and analyzed. A list of elements in the tool can be found in the supplementary material.

For this analysis, QTA scores from 2011 to 2014 for 636 audited social franchisees were extracted and analyzed using Stata version 11 and SPSS version 21.

#### Client Exit Interviews

Most MSI country programs conduct interviews annually with a random sample of family planning clients at different service delivery sites and channels. These exit interviews consist of a short, interviewer-administrated, standardized questionnaire following a client’s service visit to gather more detailed information about client demographics and socioeconomic status, services obtained, choice of contraceptive methods, and client experience. In 2013, exit interviews were conducted with 4,844 clients at 14 of the 17 national social franchise networks; Yemen did not complete exit interviews due to political instability, Zambia because the franchise had only been recently established, and Zimbabwe due to programmatic challenges that caused delays.

In the exit interviews, client satisfaction is measured by asking interviewed clients to rate their experience on a scale of 1 (very poor) to 5 (very good) on a range of questions, including friendliness and respect demonstrated by providers, waiting time, and facility cleanliness.

Poverty is measured using the Progress out of Poverty Index (PPI)^10^ in the countries where this index is available (i.e., all MSI countries with franchising except Madagascar and Zimbabwe); for MSI country programs where the PPI is not available, the Multidimensional Poverty Index is used instead.^11^ Results from the PPI enable estimation of the proportion of clients living on less than US$1.25/day and those living on less than $2.50/day, two commonly used measures of national poverty.^12^

Clients are also asked about their recent use of contraception. “Family planning adopters” are defined as clients who had not been using any modern method of family planning during the 3 months prior to their visit.

Data from exit interviews were entered at a national level into version 3.5.4 of Epi Info and then imported into version 21 of SPSS for analysis at a country level. For the analysis in this paper, 2013 client exit interview data from 13 countries were exported from SPSS and analyzed using Stata version 11.

### Data Analysis

We combined information from the routine program monitoring data, clinical quality audits, and client exit interviews to examine success of the social franchising program in achieving the 4 intended outputs under the MSI results framework: access, efficiency, quality, and equity (Table 1).^13^

Results of the MSI social franchising program are based on routine service data, clinical quality audits, and client exit interviews.

**TABLE 1.** Marie Stopes International Social Franchising Results Framework: Measures of Intended Outputs

**Intended Output**	**Definition**	**Data Source**	**No. of Countries and Years**	**Indicators**
Access	The extent to which a program ensures potential clients can reach or obtain services regardless of financial, geographic, or cultural barriers to access	Routine program monitoring data	Starting in 7 countries in 2008 and growing to 17 countries by the end of 2014	Estimated number of family planning clients
				Number of couple-years of protection (CYP)
Efficiency	How inputs (financial, human, technical) are used to produce the maximum output	Routine program monitoring data	Starting in 7 countries in 2008 and growing to 17 countries by the end of 2014	Average number of CYPs generated per social franchisee per year
Quality	The degree to which a provider or facility meets certain objectives and perceived levels or expectations of health care delivery standards	Clinical quality audit	10 countries in 2011,10 countries in 2012,15 countries in 2013,14 countries in 2014	Mean quality score of audited social franchisees
				Proportion of audited social franchisees scoring over minimum standard (score ≥80%)
		Client exit interviews	14 countries in 2013	Self-rating of overall experience
Equity	The extent to which a program ensures all potential clients have an equal or fair opportunity to obtain services.	Client exit interviews	14 countries in 2013	Family planning adopters: Proportion of family planning clients who newly adopt a modern contraceptive method (defined as not using a modern method during the 3 months prior to their visit)
		Client exit interviews	14 countries in 2013	Age: Proportion of clients under 25 years old and proportion under 20 years old
		Client exit interviews	12 countries in 2013	Poverty: Proportion of clients living below US$1.25/day and proportion living below $2.50/day

**Access** was assessed using both estimated numbers of clients and couple-years of protection (CYPs). The number of clients receiving short-acting contraceptive methods was estimated by dividing the number of commodities provided by the number of commodities needed for a full year of contraceptive protection (e.g., 13 for oral contraceptive pills; 98 for condoms; or 4 for DMPA contraceptive injections, given every 3 months). The number of clients receiving LARCs was obtained from the number of voluntary IUD and implant insertion services delivered, and the number of clients receiving voluntary permanent methods was obtained from the number of tubal ligation and vasectomy services provided. International conversion factors were used to convert the number of services and commodities provided into CYPs, a standard measure of the estimated amount of time a couple will be protected against unintended pregnancy per unit of the contraceptive method used.^14^ CYP conversion factors differ slightly from the estimates used in the “number of clients” calculation because CYP factors account for method effectiveness and wastage. (For a list of the conversion factors, see http://www.usaid.gov/what-we-do/global-health/family-planning/couple-years-protection-cyp.) Changes over time were assessed using a non-parametric test for trend across ordered groups.

**Efficiency** was measured at the national level by dividing annual countrywide CYPs (for the entire franchise network) by the number of franchisees in operation at the end of each calendar year, with the exception of the first year of a franchise’s operation. For these new country franchises, the number of franchisees at year’s end was divided by the total number of months the franchise had been operating to smooth the data during the period of largest proportional growth. Changes over time were assessed using a non-parametric test for trend across ordered groups.

**Quality** was assessed using overall clinical audit scores and overall client satisfaction scores. Raw clinical quality audit scores were compared using pairwise adjusted Wald tests. The proportion of franchisees scoring above a minimum acceptable score of 80% was compared over time using chi-square tests. Countries were treated as strata, and QTA results were weighted by the number of social franchisee outlets operating in each country at the end of the calendar year. Client satisfaction was weighted by the number of family planning clients in each country over the calendar year. The significance of multiple pairwise comparisons was adjusted using Holm’s method^15^; all *P* values presented in the results include these adjustments.

**Equity** was evaluated using client exit interview data to assess the proportion of clients who were family planning adopters (i.e., those who had not used any modern method during the 3 months preceding the service), youth, or living under US$1.25/day or $2.50/day. Results from Madagascar and Nigeria were excluded from the poverty analysis as they used non-comparable measures to the PPI (Madagascar used the Multidimensional Poverty Index and Nigeria’s PPI calculates the proportion living under the national poverty line rather than under international poverty lines). Countries were treated as strata, and franchisees served as the primary sampling unit for clustering in the analysis; exit interview results were weighted by the number of family planning clients in each country over the calendar year.

**Outcomes** were estimated using MSI’s “Impact 2” model. Full details of the model are described elsewhere^16^; in short, the model enables conversion of service data into a variety of estimated health outcomes using the best available data on country demographics, fertility, mortality, and more. To estimate the number of unintended pregnancies that will be averted, for example, method-specific failure rates are applied to modeled users of each, taking discontinuation into account. The results are then compared with the average number of pregnancies that would have occurred had the woman not been using any contraception. Other measures, such as the number of maternal deaths averted, are more complicated. Estimating the number of maternal deaths averted involves 6 point estimates from the World Health Organization of the maternal mortality ratio (MMR) for each country, modeling of the ratio’s change over time in each country, isolating the portion of maternal mortality due to live births, and combining that mortality with the estimates of unintended pregnancies averted.

For this analysis, routine program monitoring data (number of services and number of commodities) was used to estimate the number of unintended pregnancies, maternal deaths, maternal disability-adjusted life years (DALYs) lost, and costs in direct health care spending that will be averted due to services delivered by MSI’s social franchising program from 2008 through 2014.

## RESULTS

### Access

#### Number of Family Planning Clients

**FIGURE 1.**
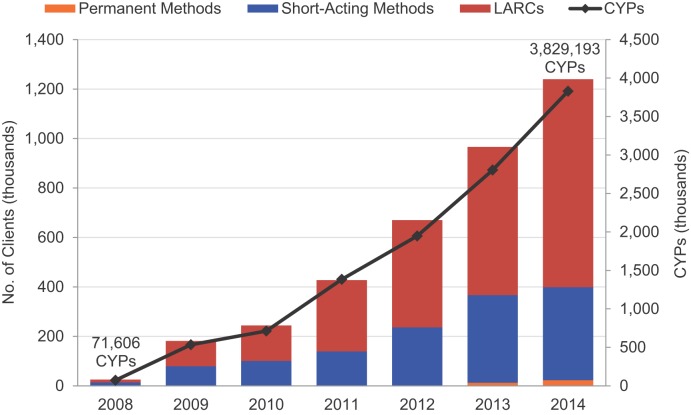
Annual Number of Family Planning Clients^a^ by Type of Contraception and Annual Couple-Years of Protection Provided by MSI Social Franchising Programs in Selected African and Asian Countries,^b^ 2008–2014 Abbreviations: CYPs, couple-years of protection; LARCs, long-acting reversible contraceptives; MSI, Marie Stopes International. ^a^ The number of family planning clients was estimated as follows: for short-acting methods, the number of commodities provided each year divided by the number of commodities needed for a full year of contraceptive protection (i.e., 13 for oral contraceptive pills; 98 for condoms; 4 for DMPA contraceptive injections, given every 3 months; these factors differ slightly from CYP conversion factors because CYP conversion factors take into account method effectiveness and wastage); for LARCs, the number of IUD and implant insertion services provided each year; and for permanent methods, the number of tubal ligation and vasectomy services provided each year. ^b^ Data are from routine program monitoring, from 7 countries in 2008 and growing to 17 countries in 2014.

In 2008, the MSI social franchise program provided voluntary family planning services to an estimated 25,335 clients. By 2014, this provision had grown to an estimated 1,239,727 clients, soaring by nearly 49-fold (Figure 1). Over the entire 7-year period, MSI reached an estimated 3,753,065 family planning clients cumulatively.

MSI social franchising programs cumulatively reached 3.75 million family planning clients over 7 years.

Clients receiving LARCs and permanent methods accounted for an estimated 50% of clients in 2008, rising significantly to 70% by 2014 (*P* = .02). The increasing number of clients was significant across all client types (*P* = .02 for short-acting method clients, LARC clients, and all clients, and *P* = .047 for permanent method clients). The vast majority of these services were for LARCs. Method-specific results can be found in the supplementary material.

68% of the social franchising clients chose LARCs.

In the Asian countries where MSI has social franchising programs, the LARCs provided were exclusively IUDs through 2011. Implant provision has been increasing in these countries since 2012 but still accounted for only 1% of LARCs in 2014. African programs initially distributed more IUDs than implants, but, overall, that pattern was reversed by 2010 (slightly earlier in West Africa, and slightly later in Southern Africa). The disparity between the two LARC methods peaked around 2012, but since then IUDs have increased as a proportion of LARCs in Africa. Still, in 2014, 75% of LARCs provided through franchisees in Africa were implants.

#### Number of CYPs

Total CYPs delivered by MSI social franchising programs increased 54-fold, from 71,606 in 2008 to 3,829,193 in 2014 (Figure 1). The trend for this increase was statistically significant (*P* = .02).

### Efficiency

The absolute number of MSI social franchisee outlets has increased over time, from 695 outlets in 7 countries at the end of 2008 to 4,070 outlets in 17 countries by the end of 2014. In 2014, the median number of social franchise outlets per national social franchise program was 225, ranging from 25 outlets in Sierra Leone to 589 outlets in Ethiopia. The next smallest franchise was Zambia, with 38 franchisees.

**FIGURE 2.**
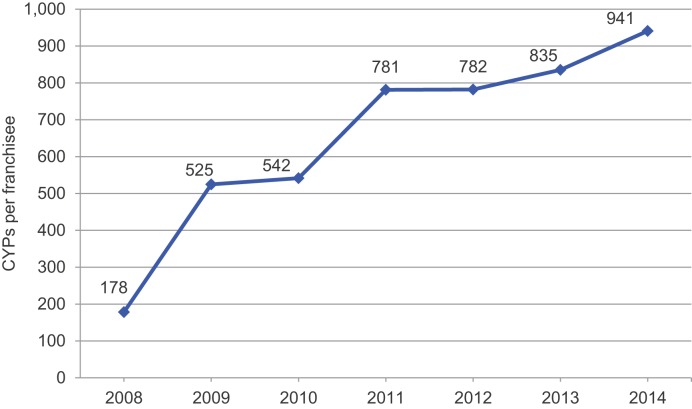
Annual Couple-Years of Protection Provided per MSI Franchisee,^a^ 2008–2014 Abbreviations: CYPs, couple-years of protection; MSI, Marie Stopes International. ^a^ Data are from routine program monitoring, based on 695 social franchisee outlets in 7 countries at the end of 2008 and growing to 4,070 outlets in 17 countries by the end of 2014.

While the number of franchisees has grown substantially, the increase in outlet numbers did not account solely for the increase in client numbers or CYPs. Efficiency, measured by CYPs generated each year by each social franchisee outlet, has also increased over time, from an average of 178 CYPs per outlet in 2008 to 941 CYPs per outlet in 2014 (*P* = .02) (Figure 2). Increases in efficiency have accounted for an estimated 61% of the growth in total CYPs, while increases in the number of franchisees have accounted for the remaining 39%. Nearly all (98%) of the increase in efficiency was due to rising numbers of family planning clients per franchisee; provision of longer-acting contraceptive methods, which contribute higher CYPs per contraceptive unit, accounted for little more than 2% of growth in efficiency.

Improved efficiency of social franchisees has accounted for 61% of the growth in couple-years of protection provided over time.

### Quality

#### Mean Quality Score

The mean quality score among audited clinical social franchisees has also increased significantly over time (*P*<.002); among the 61 franchisees audited in 2011, the mean score was 78.5 (95% confidence interval [CI] = 73.5, 83.5), increasing to 87.9 (95% CI = 86.5, 89.3) among the 200 franchisees assessed in 2014 (Table 2). Quality scores in adjacent years were not significantly different, except between 2012 and 2013. The overall significance over time, however, held whether scores for all audited social franchisees were included or only scores from social franchisees in which there was a national social franchise program operating during all 4 years being assessed (n = 8 countries).

**TABLE 2.** Quality Audit and Client Satisfaction Scores of Selected Marie Stopes International Social Franchisee Outlets, 2011–2014

	**2011 (N=61)**	**2012^a^ (N=164)**	**2013 (N=211)**	**2014 (N=200)**
Overall quality score, mean (95% CI)	78.5 (73.5, 83.5)	80.8 (79.4, 82.3)	86.6 (85.3, 88.0)	87.9 (86.5, 89.3)
Proportion of audited outlets scoring higher than minimum standard score of 80%, % (95% CI)	39.8 (20.9, 58.8)	58.7 (49.5, 67.9)	77.4 (71.7, 83.1)	84.1 (78.5, 89.6)
Client satisfaction score,^b^ mean (95% CI)			4.51 (4.46, 4.56)	

Quality scores are from clinical quality audits while client satisfaction scores are from client exit interviews.

a The program in Pakistan used a slightly amended scoring methodology for clinical audits in 2012.

b Total possible client satisfaction score was 5.

#### Proportion Scoring Above Minimum Standard

In 2011, 39.8% of audited social franchisee outlets scored higher than the minimum standard score of 80%. The proportion scoring higher than the minimum standard score increased to 84.1% in 2014 (Table 2). Comparisons between years were similar to those of the raw scores, with no significant difference detected between 2011 and 2012 or between 2013 and 2014. But the overall increase in scores was statistically significant between the first two years and final two years (*P*<.001).

#### Client Satisfaction

In 2013, the average weighted client satisfaction score among outlets in 14 countries was 4.51 (95% CI = 4.46, 4.56), out of a total possible score of 5 (Table 2).

### Equity

#### Age

Among the MSI franchise programs in 14 countries that conducted client exit interviews in 2013, the overall proportion of clients aged 15–19 years was 5.0% (95% CI = 3.9, 6.1), and the overall proportion aged 15–24 years was 26.1% (95% CI = 23.8, 28.4) (Table 3). The highest proportion of clients aged 15–24 years were in Mali (42.9%, 95% CI = 36.3, 49.5) and Uganda (41.4%, 95% CI = 25.5, 57.3), and the lowest proportion in Vietnam (8.6%, 95% CI = 5.4, 11.8) and Pakistan (11.0%, 95% CI = 9.7, 12.3) (see supplementary material).

About one-quarter of social franchisee clients were 15–24 years old.

**TABLE 3.** Equity in Access to Family Planning Through Marie Stopes International Social Franchising Programs in Selected Countries, 2013

**Equity Domain**	**%, (95% CI)**
Age of clients	
15–19 years	5.0 (3.9, 6.1)
15–24 years	26.1 (23.8, 28.4)
Poverty level of clients^a^	
Living on under US$1.25/day	15.1 (13.8, 16.4)
Living on under US$2.50/day	57.4 (54.9, 60.0)
Family planning adopters^b^	
Newly adopted a modern contraceptive method	40.7 (37.4, 44.0)

Data are based on client exit interviews in 14 countries (Ethiopia, Ghana, India, Kenya, Madagascar, Malawi, Mali, Nigeria, Pakistan, Philippines, Senegal, Sierra Leone, Uganda, and Vietnam), except for the “poverty” indicator, which excludes results from Madagascar and Nigeria because they used non-comparable measures of poverty than the other 12 countries.

a Measured using the Progress out of Poverty Index (PPI).^13^

b Defined as modern contraceptive method clients who had not been using a modern method during the 3 months prior to their visit.

#### Poverty

In 2013, the overall proportion of clients living on under US$1.25 a day was 15.1% (95% CI = 13.8, 16.4), and the overall proportion living on under $2.50 a day was 57.4% (95% CI = 54.9, 60.0) (Table 3). The highest proportion of clients living on under $1.25 a day was in Mali (39.6%, 95% CI = 32.3, 46.8) and Sierra Leone (32.2%, 95% CI = 25.2, 39.1), but in both countries, the proportion of clients living on under $1.25 a day was still lower than the proportion of the national population living on under $1.25 a day. The countries with the lowest proportion of clients living on under $1.25 a day were Ghana (2.1%, 95% CI = 0.6, 3.6) and Vietnam (4.9%, 95% CI = 3.3, 6.6%). More country-specific information can be found in the supplementary material.

Social franchisees reached more clients living in near poverty than in extreme poverty.

#### Family Planning Adopters

In 2013, 40.7% of family planning clients reported they had not been using a modern method during the 3 months prior to their visit (i.e., that they were family planning adopters) (95% CI = 37.4, 44.0) (Table 3). In 5 of the 14 countries, more than 50% of family planning clients reported they were new family planning adopters (see supplementary material). Of the family planning adopters, 46.1% (95% CI = 40.9, 51.2) reported having never previously used family planning, including traditional methods.

### Outcomes

Using the MSI Impact 2 model, family planning services provided by MSI’s social franchising program between 2008 and 2014 will avert an estimated 4,958,000 unintended pregnancies and 7,150 maternal deaths. The services will also avert an estimated 6,986,500 maternal DALYs lost, and save US$197,812,500 in direct health care spending.

## DISCUSSION

Clinical social franchising through an existing network of private providers offers a promising model to rapidly scale-up access to voluntary family planning information and services, while simultaneously improving quality of services. Such scale-up is needed to achieve the visionary goal set at the London Family Planning Summit to reach 120 million more women and girls with contraception.

Clinical social franchising offers a promising model to rapidly scale-up access to family planning services.

In just 7 years, MSI has successfully expanded its social franchising program to more than 4,000 social franchisees across 17 countries, providing voluntary family planning services to almost 3.75 million women cumulatively, many of them poor. Furthermore, two-fifths of modern contraceptive users had not used family planning recently, and nearly half of these women had never used family planning at all—these users are a key group in addressing unmet need. A substantial 68% of the women reached by MSI social franchising opted for voluntary LARCs. At the same time, MSI was able to greatly increase the efficiency of franchisees as measured by the number of CYPs generated each year by each franchised outlet. By expanding the number and productivity of franchisees, MSI has increased its reach to clients by almost 5000% over 7 years. A number of specific country-level approaches, such as introduction of new services including postpartum IUD insertions, have contributed to the improved quality and expansion of services (Box).

**BOX. Strengthening Social Franchising: Case Examples**

Several of MSI’s national social franchising programs have improved the quality and expanded the range of services that franchisees provide through different approaches. Some examples include:

- **Improving quality:** The social franchise network in Pakistan had the highest level of clinical quality in 2013 of all MSI national social franchise networks. A centralized procurement system that ensures control over commodities and supplies may be a contributing factor. Additionally, the network has invested heavily in supportive supervision, with a ratio of 1 field supervisor to every 10 franchisees.
- **Introducing new services:** In Kenya and Nigeria, many social franchisees provide obstetric services including antenatal care and safe delivery. Recently, these franchisees have been trained in postpartum IUD insertion services. Providing IUDs during the postpartum period further expands contraceptive choice for women at a time of high unmet need. This intervention has resulted in increased access to IUDs as part of a broad range of family planning methods available. The external QTA conducted among 4 providers in Kenya found an average score above the minimum benchmark, suggesting strong retention of skills.
- **Expanding choice:** In Uganda, social franchisees expanded contraceptive choice by addressing supply- and demand-side constraints. On the supply side, social franchisees received counseling training on all contraceptive methods, qualified providers received training on LARC and permanent method service provision while referral pathways were established for providers not able to provide permanent methods, and providers were supplied with equipment and commodities. Demand-side constraints were addressed through a voucher program that reduced financial barriers and facilitated access to voluntary family planning services for poor women. In 2013, 40% of Uganda’s social franchise clients were “family planning adopters” (i.e., they had not been using a modern method during the prior 3 months) and 61% lived on less than US$2.50/day.

### Impact on Population-Level Outcomes

Through these expansions, we estimate that MSI’s program will avert nearly 5 million unintended pregnancies and approximately 7,150 maternal deaths, and lead to estimated savings in direct health care costs of over US$197 million. A previously published study found that MSI’s social franchise program in Pakistan increased the overall contraceptive prevalence rate by almost 20% in intervention districts compared with control districts.^17^ Together, these findings provide evidence of the positive impact that social franchising programs can have on population-level health outcomes. Further evidence of health outcomes from social franchising is expected to be generated from a randomized controlled trial currently being conducted through the impact evaluation of the African Health Markets for Equity program,^18^ as well as one planned by MSI of its own franchising activities in Pakistan.

MSI’s social franchising program averted nearly 5 million unintended pregnancies and about 7,000 maternal deaths, saving over $197 million in direct health care costs.

### Expanding Contraceptive Choice

In addition to providing family planning services to those with unmet need, expanding access can be viewed through the lens of providing additional contraceptive method choices to users. LARCs and permanent methods are safer and more reliable for women than many short-acting methods, but these clinical methods require more training than short-acting methods and thus are seen as more difficult to provide. Furthermore, community myths and misconceptions about LARCs and permanent methods, as well as the associated upfront costs for clients, compound the issue.^19^^-^^21^ A recent technical meeting highlighted the question of whether social franchising could increase access to LARCs and permanent methods among family planning adopters.^7^ The 7 years of data analyzed in this paper clearly demonstrate that franchising can increase access to LARCs. By 2014, 68% of methods provided by MSI social franchising programs were LARCs, and nearly half of MSI’s family planning clients who had not been recently using family planning opted for LARCs. We are seeking additional information on the proportion of providers who had lacked skills to offer LARCs prior to being franchised, but initial reports suggest a sizable majority of providers gained new skills in family planning service delivery by joining a franchise network.

While there is strong evidence for the ability of social franchising to offer LARCs, there remains less clarity for provision of permanent methods. Less than 25% of MSI’s national social franchise networks offered permanent methods in 2014, and delivery of these methods remains low, accounting for less than 2% of overall client numbers in 2014. In addition, in many franchise networks, MSI deliberately recruited lower-level providers, such as midwife- and nurse-led clinics, based on the belief that such franchisees are better able to reach poorer and more underserved clients than doctor-led clinics.^5^ While focusing on lower-level providers may improve equity in service delivery, these providers are commonly unable to offer permanent methods under national regulatory frameworks. Further investigation of strategies, such as task sharing or strengthened referral mechanisms, to safely increase the number of social franchisees that are able to provide voluntary permanent methods is needed.^22^

Social franchising clearly improved access to LARCs, but other strategies may be needed to improve the ability of social franchisees to provide permanent methods.

### Improving Quality

During scale-up, it is critical to ensure that the services provided are, and remain, of high quality. We recorded high levels of client satisfaction through client exit interviews. We also, encouragingly, found a significant increase over time in external quality audit scores, at the same time that MSI’s social franchise networks were rapidly expanding. However, ensuring quality of all outlets across large, geographically dispersed networks remains a challenge and requires major investment of resources. MSI is currently improving its quality monitoring systems through use of a new quality assurance framework, which emphasizes ongoing monitoring of quality to reduce reliance on an annual audit cycle. The new framework includes using information and communications technology to continually gather and analyze data, feed results back to franchisees, and support a supervision process tailored to individual outlet needs. Beginning in 2015, external audit resources will be aligned to emphasize quality assurance in sites that have the most variability or that have lagged in quality increases over the past several years. Additionally, a number of organizations are engaged in a global discussion around measuring the process quality in franchised facilities and gaining a more rigorous understanding of other dimensions of quality such as client satisfaction.

### Bridging Equity Gaps

Although MSI has successfully scaled-up access to family planning services through social franchising, it is important to also consider the types of clients being reached to ensure equitable access to services. In 6 of the 14 MSI national social franchise programs that conducted exit interviews in 2013, the proportion of clients aged 15–24 years was significantly larger than the proportion of family planning demand in the national married population from those aged 15–24 (see supplementary material), pointing to MSI’s ability to reach **younger populations**, a group which is often underserved.^23^ Still, only a relatively small proportion of MSI’s clients were under 20 years, a group that, as they become sexually active, is critical to access with services to prevent pregnancy at a young age and the associated health and social risks.^23^ However, the social franchisee model, including its associated fee structure, may not be the most appropriate channel to reach this demographic without associated mechanisms to reduce financial or other barriers to access.

We found mixed results in reaching **poor clients**; while 57% of MSI social franchising clients lived on under US$2.50 a day—the median poverty line in all but the 15 poorest countries^12^—only 15% of clients lived on less than $1.25 a day. No country programs had a significantly larger proportion of clients living on less than $1.25 a day than the proportion of the national population living on under $1.25 a day. But we had better results with clients living on under $2.50 a day; several countries had franchise networks with significantly more clients living on under $2.50/day than the proportion of the national population living on under the same amount. Details can be found in the supplementary material.

One country with substantially more near-poor clients than in the national population was Pakistan. In Pakistan, only providers in periurban and rural areas are selected to be franchised. Additionally, a network of community mobilizers builds demand in communities, and many of these mobilizers provide vouchers to those unable to afford family planning. While such characteristics could be associated with success in reaching those living in poverty, more evidence is needed of successful programs. There are ongoing debates about the ability of clinical social franchising to reach the poorest, particularly in the absence of financing mechanisms such as voucher programs and publicly financed health care^6^^,^^24^^,^^25^; only 6 of the 17 social franchisee programs included in this analysis were operating voucher programs during the time frame of this analysis. In addition, many social franchisees may not be located in areas where the majority of the population is poor, or, if they are, the poorest clients may still be unable to access these services due to actual or perceived costs and may instead be accessing private health care through the informal sector.^26^ Further analysis of other clinical social franchise programs, including their use of various health financing mechanisms and use of national wealth indices to measure relative wealth, would greatly assist in understanding the current situation before formulating approaches to better reach the poorest, whether through social franchising or other service delivery mechanisms such as mobile outreach services.^27^

While there were mixed results in reaching particular groups of clients with high unmet need—reaching some in near poverty but few in extreme poverty, and successfully serving youth aged 20–24 but few under 20—MSI’s social franchising program was more consistently able to reach **women who had not been using family planning recently** and those who had **never used family planning**. Some clients have not used family planning recently because they were not sexually active at the time or because they recently changed their fertility intentions (i.e., they had been trying to get pregnant). However, many who have not been using contraception actually do not want children in the near future and have a real unmet need for family planning. Providing family planning services to those who have not used contraception for a period longer than 3 months is therefore key to addressing unmet need. In addition, serving such family planning adopters suggests social franchisees are not solely competing with other providers for existing clients. In some countries, over 70% of MSI’s social franchising family planning clients were family planning adopters.

MSI's social franchising program successfully reached women who had not been using contraception recently, including those who had never used it.

### Limitations

There are some limitations to the analyses discussed above. Client numbers were estimated from number of services and commodities provided, which may lead to over- or underestimates of true client numbers. Client numbers may also be underestimated as we did not include clients who had received family planning services such as counseling or the removal of IUDs or implants but who did not also choose to receive a modern contraceptive method at the same time. It is possible that franchisee reports of provision of short-acting methods are underreported compared with long-acting methods, as several social franchising programs include voucher programs aimed at reducing barriers to accessing more expensive LARCs; thus, there are direct incentives to report all LARCs provided. These limitations will generally lead to under- rather than overestimation of the true client numbers, and thus are likely conservative in their potential bias.

Aspects of the outputs assessed could not be included. For example, efficiency measures based on cost-effectiveness or access measures based on geographical location or provider discrimination were unable to be included at this stage. We are working to add such indicators to subsequent evaluations.

The location of several franchise networks also led to imperfect data collection. Due to the fragile security situation in Yemen, the Ebola outbreak in Sierra Leone, and a temporary suspension in operations in Uganda, the quality and equity results contain several gaps, and thus are not completely representative of MSI’s entire franchising program. With clinical quality audits, there have been slight amendments made over time to the sampling and checklists used. These changes were intended to strengthen the audits based on lessons from previous years but could reduce comparability between years. Client satisfaction questions are often affected by courtesy bias in responses, and collection of true satisfaction could be imperfect as well.

Health outcomes were modeled rather than directly assessed. These estimates were based on the best available data but cannot provide the accuracy of other methods. Efforts were made to minimize the occurrence and impact of these limitations, but many of them reflect the reality of the environments these franchise programs operate in.

## CONCLUSION

Through social franchising, MSI reached almost 3.75 million women with voluntary family planning services, many of whom were considered new contraceptive users, and a substantial 68% chose LARCs. At the same time as rapidly expanding its network, MSI increased efficiency of its outlet operations, significantly increased clinical quality, obtained high levels of client satisfaction, and reached a substantial proportion of young women and women living on under $2.50/day. Taken together, these results reflect the ability of clinical social franchising to rapidly scale-up global access to voluntary family planning services in the coming years and to make a substantial contribution to achieving the FP2020 goal.

## Notes

**Peer Reviewed**
